# Supplementary figures and images for: MAVS mediates a protective immune response in the brain to Rift Valley fever virus
Source: PLoS Pathog. 2022 May 18;18(5):e1010231. doi: 10.1371/journal.ppat.1010231 (PMC9154093; doi:10.1371/journal.ppat.1010231)

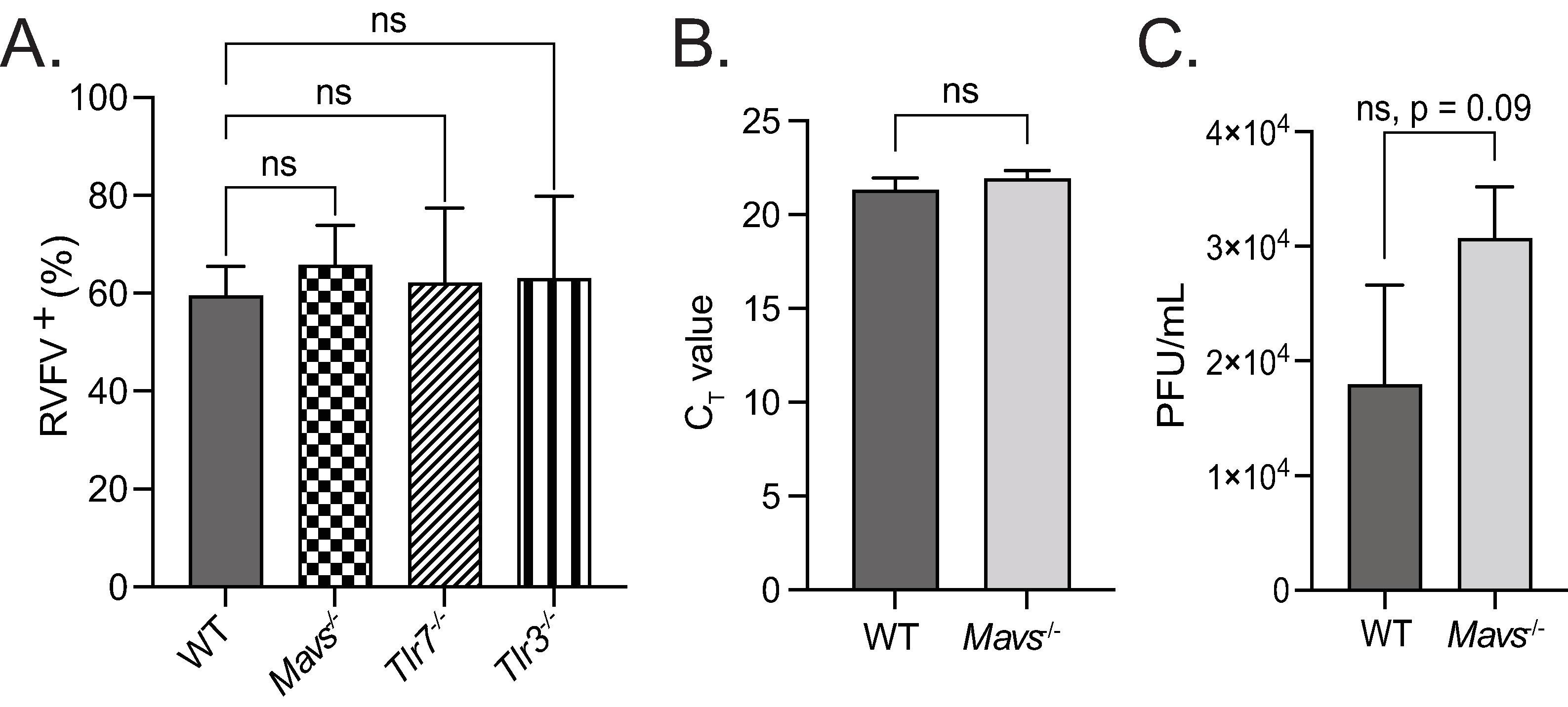

Supplement: S1 Fig — Primary microglia derived from WT or the indicated genetically deficient mice were infected with RVFV MP-12 and the percentage of cells positive for RVFV was assessed by flow cytometry (A) or levels of RVFV genomic RNA were assessed by qPCR (B). Primary microglia derived from WT or Mavs-/- mice were infected with RVFV MP-12, supernatants collected at 24 hours post-infection, and viral titers in supernatants assessed by plaque assay (C). (TIF) [file ppat.1010231.s001.tif]

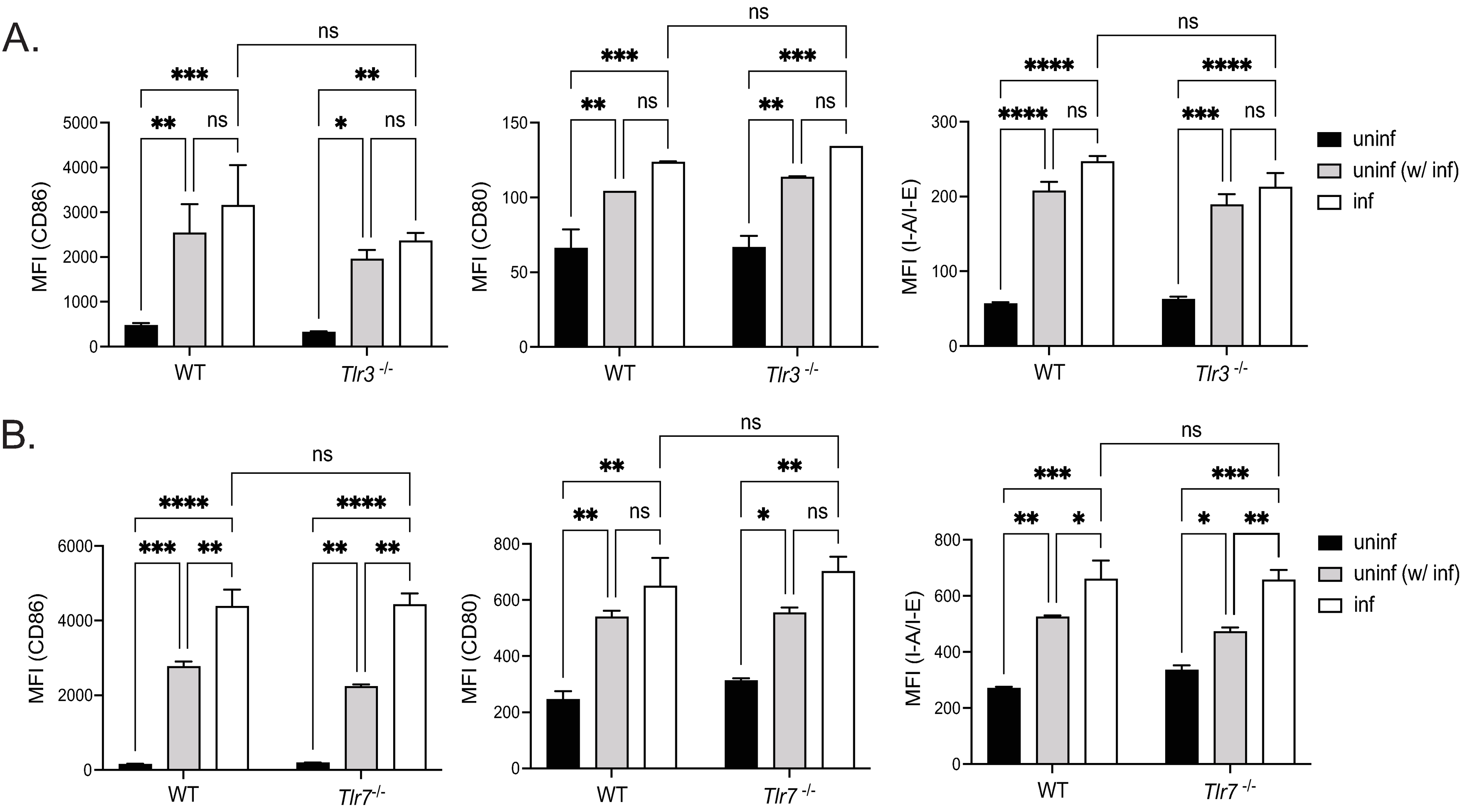

Supplement: S2 Fig — Microglia derived from WT or Tlr3-/- (A) or Tlr7-/- (B) mice were infected with RVFV MP-12 and at 18–24 hours post-infection, cells were harvested for flow cytometry. The expression levels of the indicated activation markers were assessed on uninfected cells (black bars), uninfected cells in culture with infected cells (uninf (w/ inf), gray bars), and infected cells (inf, white bars) and shown as the mean fluorescence intensity of the indicated activation markers. Data are shown as the mean +/- SD *p <0.05, **p<0.01, ***p<0.001, ****p<0.0001 (TIF) [file ppat.1010231.s002.tif]

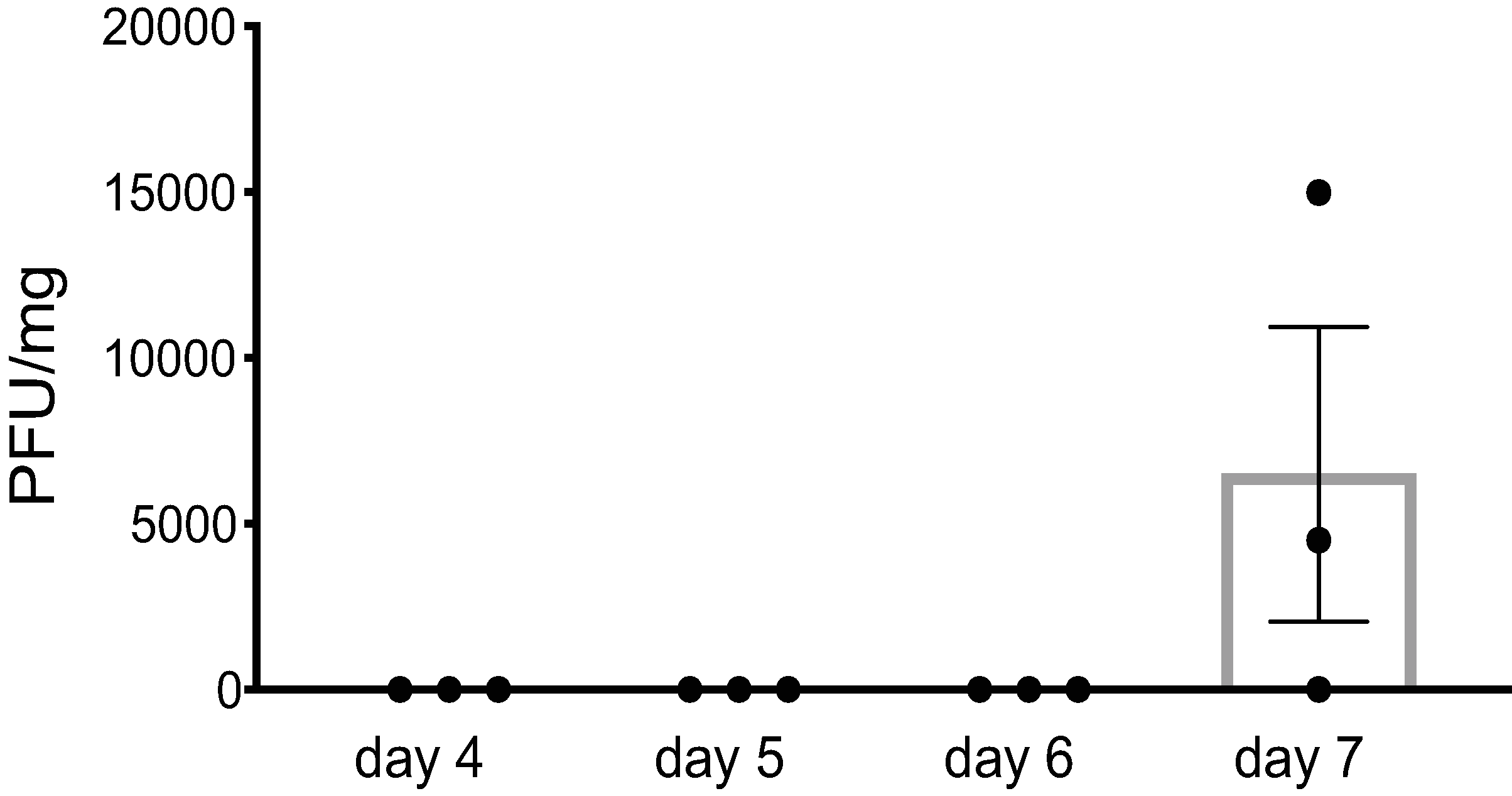

Supplement: S3 Fig — WT mice were infected intranasally with 5x10^5^ PFU RVFV MP-12 and brains were harvested on the indicated day post-infection for viral quantitation. (TIF) [file ppat.1010231.s003.tif]

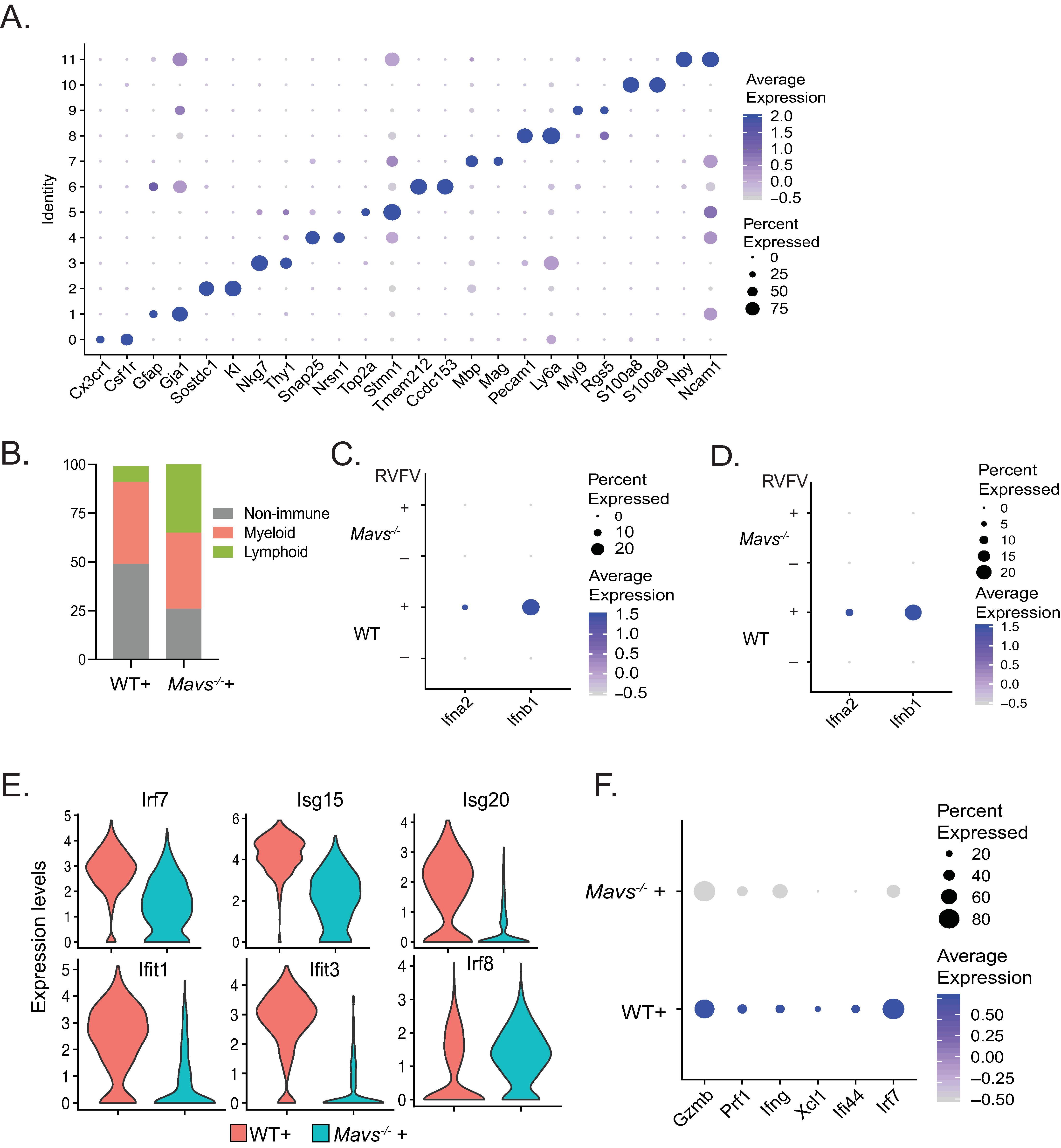

Supplement: S4 Fig — Gene markers for specific cell types defining the cell clusters in Fig 5A (A). The distribution of cell types within female infected brains (B). Expression of type I IFNs within astrocytes (Fig 5A cluster 1, C) and oligodendrocytes (Fig 5A cluster 7, D). Violin plots depicting the relative expression of selected genes within microglia from female infected brains (E). Dot blot depicting gene expression within lymphocytes from female infected brains (F). (TIF) [file ppat.1010231.s004.tif]

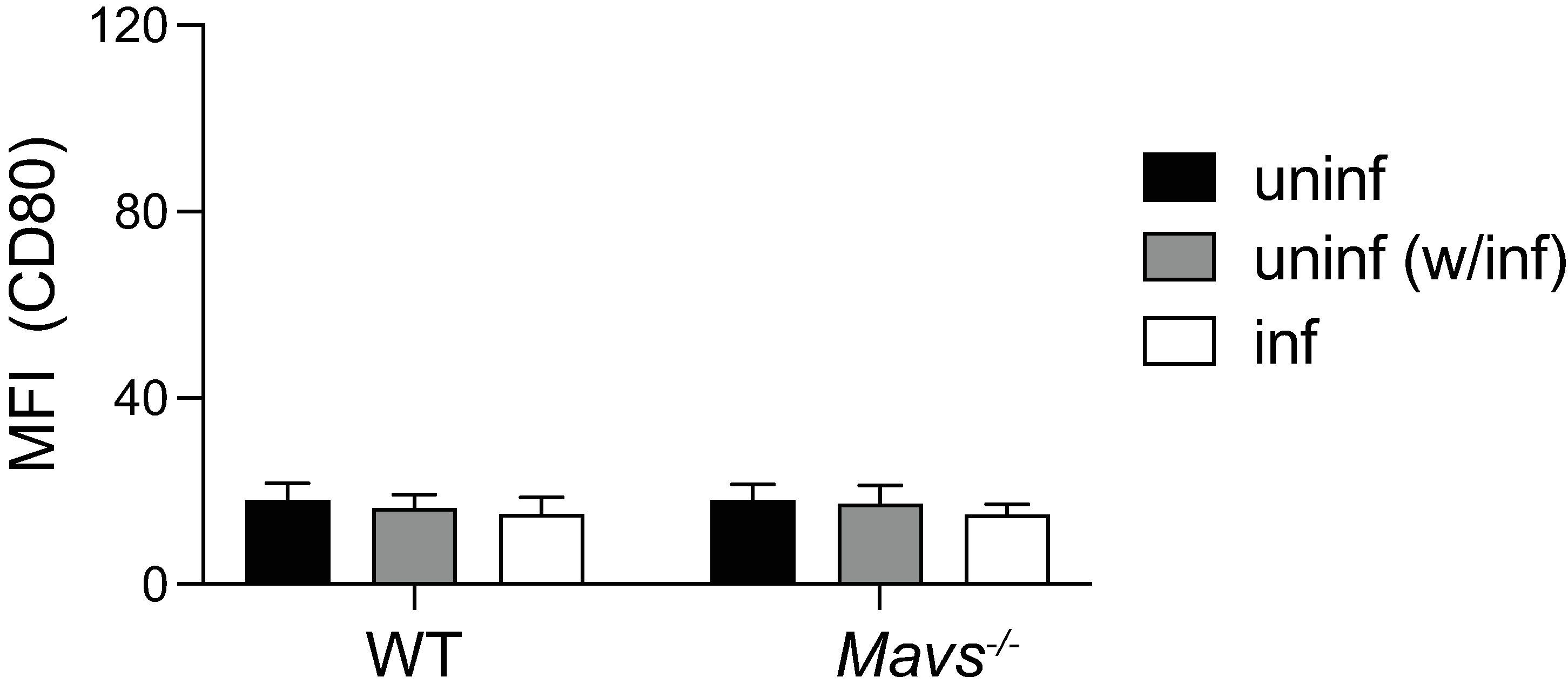

Supplement: S5 Fig — Mean fluorescence intensity of CD80 on microglia isolated from the brains of WT and Mavs-/- mice, +/- RVFV infection. (TIF) [file ppat.1010231.s005.tif]

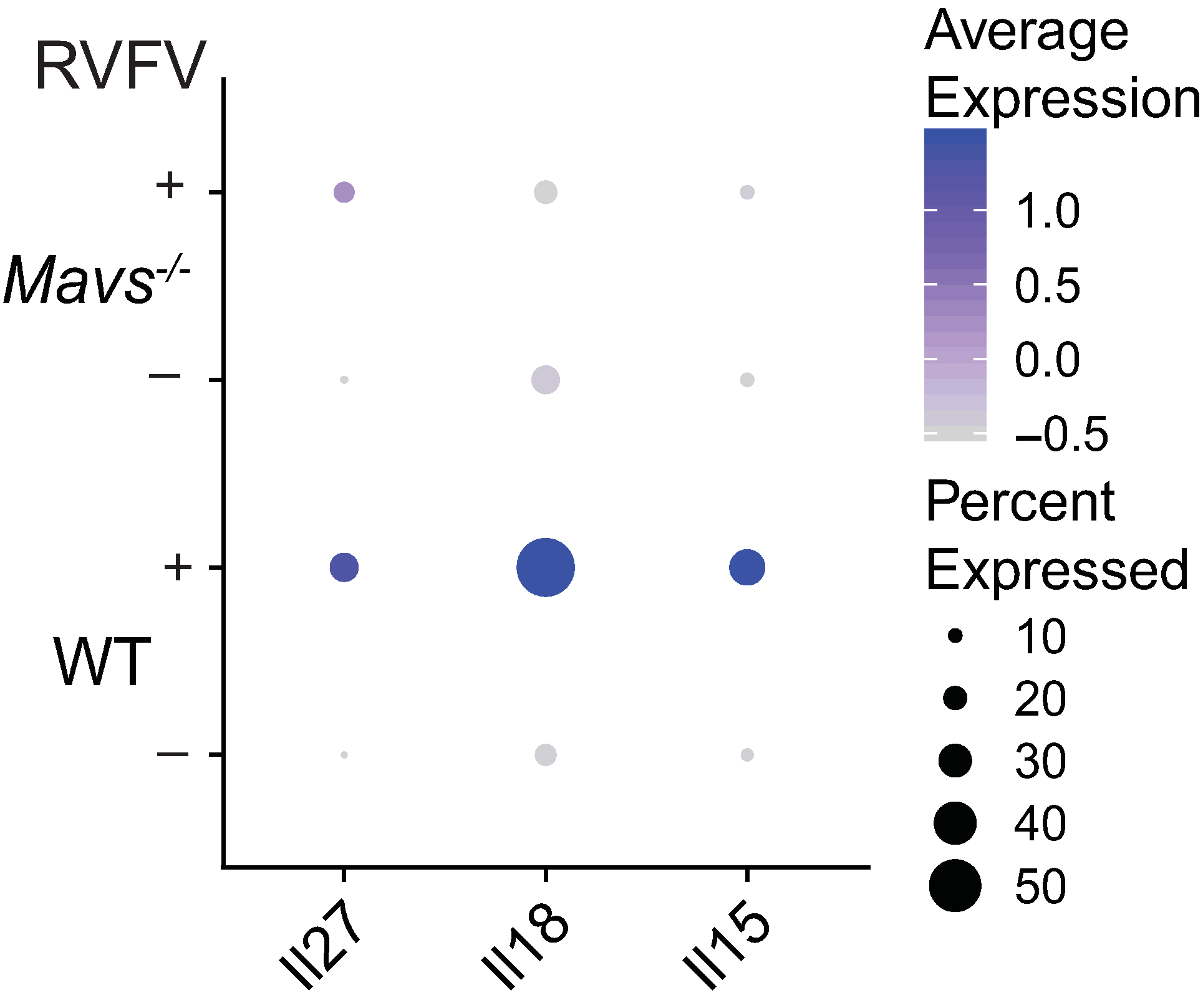

Supplement: S6 Fig — (TIF) [file ppat.1010231.s006.tif]
